# Exercise in children with joint hypermobility syndrome and knee pain: a randomised controlled trial comparing exercise into hypermobile versus neutral knee extension

**DOI:** 10.1186/1546-0096-11-30

**Published:** 2013-08-14

**Authors:** Verity Pacey, Louise Tofts, Roger D Adams, Craig F Munns, Leslie L Nicholson

**Affiliations:** 1 Physiotherapy Department, The Children’s Hospital at Westmead, Sydney, Australia; 2 Kids Rehab, The Children’s Hospital at Westmead, Sydney, Australia; 3 Department of Endocrinology, The Children’s Hospital at Westmead, Sydney, Australia; 4 Discipline of Biomedical Sciences, The University of Sydney, Sydney, Australia; 5 Discipline of Pediatrics and Child Health, The University of Sydney, Sydney, Australia; 6 Discipline of Physiotherapy, The University of Sydney, Sydney, Australia

**Keywords:** Hypermobility, Knee pain, Physiotherapy, Intervention, Joint hypermobility syndrome, Ehlers-Danlos syndrome, Exercise, Randomised controlled trial, Hyperextension, Range of motion

## Abstract

**Background:**

Knee pain in children with Joint Hypermobility Syndrome (JHS) is traditionally managed with exercise, however the supporting evidence for this is scarce. No trial has previously examined whether exercising to neutral or into the hypermobile range affects outcomes. This study aimed to (i) determine if a physiotherapist-prescribed exercise programme focused on knee joint strength and control is effective in reducing knee pain in children with JHS compared to no treatment, and (ii) whether the range in which these exercises are performed affects outcomes.

**Methods:**

A prospective, parallel-group, randomised controlled trial conducted in a tertiary hospital in Sydney, Australia compared an 8 week exercise programme performed into either the full hypermobile range or only to neutral knee extension, following a minimum 2 week baseline period without treatment. Randomisation was computer-generated, with allocation concealed by sequentially numbered opaque sealed envelopes. Knee pain was the primary outcome. Quality of life, thigh muscle strength, and function were also measured at (i) initial assessment, (ii) following the baseline period and (iii) post treatment. Assessors were blinded to the participants’ treatment allocation and participants blinded to the difference in the treatments.

**Results:**

Children with JHS and knee pain (n=26) aged 7-16 years were randomly assigned to the hypermobile (n=12) or neutral (n=14) treatment group. Significant improvements in child-reported maximal knee pain were found following treatment, regardless of group allocation with a mean 14.5 mm reduction on the visual analogue scale (95% CI 5.2 – 23.8 mm, p=0.003). Significant differences between treatment groups were noted for parent-reported overall psychosocial health (p=0.009), specifically self-esteem (p=0.034), mental health (p=0.001) and behaviour (p=0.019), in favour of exercising into the hypermobile range (n=11) compared to neutral only (n=14). Conversely, parent-reported overall physical health significantly favoured exercising only to neutral (p=0.037). No other differences were found between groups and no adverse events occurred.

**Conclusions:**

Parents perceive improved child psychosocial health when children exercise into the hypermobile range, while exercising to neutral only is perceived to favour the child’s physical health. A physiotherapist prescribed, supervised, individualised and progressed exercise programme effectively reduces knee pain in children with JHS.

**Trial registration:**

Australia & New Zealand Clinical Trials Registry; ACTRN12606000109505.

## Background

Generalised joint hypermobility (GJH) is prevalent in 27.5% of girls and 10.6% of boys of mixed races in the United Kingdom [1] and is diagnosed when greater than normal physiological range of motion is evident in multiple joints. The prevalence of GJH in children varies across populations, due to differing methodologies and ethnicities, with rates varying from 12% of Turkish adolescents [2], 16% of Egyptian children [3], 28% of Chinese adolescents [4], 35% of Italian school-aged children [5] and 59% of Indian children [6]. While children with GJH can be asymptomatic [7], reports of musculoskeletal symptoms in hypermobile individuals are increasing [8] and children with GJH are at greater risk of developing chronic pain [9]. Knee pain is the most common musculoskeletal complaint in these children [8].

In the presence of chronic joint pain, or in conjunction with multi-system involvement of the skin, eyes, or cardiovascular system, hypermobile individuals meet the diagnosis of Joint Hypermobility Syndrome (JHS) using the Brighton criteria [10]. Children with JHS and pain have reduced physical activity and participation in functional childhood tasks such as helping round the home or riding a bike [11].

In conjunction with GJH, an individual with JHS may present with other signs and symptoms including recurrent joint dislocations or subluxations, chronic pain, marfanoid habitus, stretchy skin, varicose veins or organ prolapses. This condition can only be diagnosed following the exclusion of other known heritable connective tissue disorders [10]. Recently recognised as the same entity as Ehlers-Danlos Syndrome hypermobile type [12], JHS is now a better described condition. Current expert opinion is that physiotherapy should be the first line of treatment for knee pain in JHS [13].

At present, there is relatively little research evaluating the effectiveness of physiotherapy in reducing pain in hypermobile individuals. One cohort study [14] supports the efficacy of a lower limb closed-chain strengthening exercise programme progressed weekly in a pre-determined manner for adults with JHS, although the range in which these exercises were performed was not reported or monitored. Outcome measures of proprioception, balance, muscle strength, quality of life and pain perception all significantly improved following the single session with a physiotherapist and subsequent 8 week home-based exercise programme. This study suggests that a standardised time-contingent, progressive exercise programme undertaken with minimal supervision can assist in reducing knee pain in adults with JHS.

To date, one randomised controlled trial (RCT) has been published assessing the effectiveness of 4-6 weeks of individualised supervised physiotherapy sessions in the management of children with JHS [15]. This study compared a targeted exercise programme aimed at improving motion control of symptomatic joints through their entire range of motion with a general exercise programme including tasks such as step-ups and shuttle runs. In both groups, exercises were progressed as children gained competency in the skill. Both groups showed significant improvements in pain. No difference was found between the two programmes in reducing pain and improving function [15].

A problem for researchers trying to link intervention with theory is that the cause of joint pain in hypermobile individuals remains unknown [16]. Repetitive soft tissue microtrauma at the end of range is one possibility [17]. Recent studies of children with JHS reveal knee joint proprioceptive and muscle strength deficits [18] and reduced knee flexion throughout the gait cycle [19]. Weightbearing with knees hyperextended, together with reduced motion control arising from poor proprioceptive acuity, and reduced muscle strength and endurance to control this movement, may cause repetitive microtrauma and abnormal loading of the knee joint, resulting in knee pain.

In addressing these impairments, two paradigms for intervention may be implemented. The first involves avoiding exercise into the hypermobile range, to minimise repetitive end of range microtrauma. This paradigm views the hypermobile range as “abnormal” and implies that the patient should learn to avoid it in activities of daily living. The second paradigm regards the hypermobile range as “normal” for these patients. While biomechanically disadvantageous, this is the range in which hypermobile patients function during daily activities such as walking, and consequently where the most stability and control is required. Hence intervention should include strengthening and motion control within this hypermobile range.

While the evidence base for the role of physiotherapy in JHS management remains limited, current expert opinion recommends physiotherapy to improve dynamic stability of hypermobile joints [20] suggesting exercises should be performed into the full hypermobile range [21]. Previous reports in the literature did not state a specific range in which to exercise [14], but actively limited patients moving hypermobile joints into end of range [22]. No published evidence supports the hypothesis that exercises should be performed into the full hypermobile range.

Therefore, the present study aimed to determine if a supervised, individualised and competency-based progressive physiotherapy programme focused on improving strength and control around the knee joint is effective in reducing knee pain in hypermobile children, and whether these exercises are more, equivalently, or less effective when performed into knee hyperextension range, compared to performing them only to neutral knee extension.

## Methods

### Trial design

A single-centre, double-blind randomised controlled trial comparing two variations of an exercise program in parallel groups for children and adolescents with generalised joint hypermobility and knee pain was conducted in a tertiary hospital setting in Sydney, Australia.

### Setting and participants

Children with knee pain referred to The Children’s Hospital at Westmead’s Physiotherapy, Sports Medicine, Orthopaedic Knee, Connective Tissue Dysplasia and Rheumatology clinics between January 2007 and February 2011, were screened for eligibility by the treating clinician. Participants with a Beighton score [23] of ≥5/9 and >10° knee hyperextension were informed of the study. Any volunteers with a history of previous knee or patella dislocations, any current acute knee pathology or ligamentous insufficiency were excluded from participation. Participants were initially eligible if aged 12-16 years, but following difficulties with recruitment, ethical approval was granted to increase the age range to 7-16 years. All outcome measures remained suitable for use with the younger children, and their ability to undertake an exercise programme has been shown to be effective in the one previous RCT with children with JHS [15]. Thirty percent of children with JHS aged less than 10 years have chronic joint pain [24]. The Human Ethics Committee of both the Children’s Hospital at Westmead and The University of Sydney granted ethical approval for this study.

### Baseline assessment and outcome measures

An initial medical assessment was performed by a physician from the Connective Tissue Dysplasia Clinic. This assessment included the Beighton score, height, weight and full medical history, and confirmed eligibility for the trial. The diagnosis of JHS was assessed using the Brighton criteria [10] although this was not one of the inclusion criteria and it has not been validated for use in children to date. All participants were referred for routine echocardiography. Participants with three or more fractures were referred for bone density testing, and participants with a history of visual problems other than refractory errors were referred for ophthalmological review. These investigations were undertaken to exclude children who may have other known heritable connective tissue disorders such as Marfan’s syndrome, Ehler’s Danlos Syndrome (vascular type) or Osteogensis Imperfecta [25]. Eligible patients were recruited at the initial assessment by the physician and written, informed parental/patient consent/assent was obtained.

A physician or physiotherapist from the Connective Tissue Dysplasia Clinic assessed all outcome measures on three occasions; at inception, a minimum of 2 weeks following enrolment in the trial prior to commencing physiotherapy, and following completion of the 8 week physiotherapy programme. The second assessment was undertaken to determine the effect of recruitment, and the final assessment to measure the effectiveness of the intervention.

The primary outcome measure was child-reported average and maximum knee pain over the last week, recorded on a visual analogue scale (VAS). The VAS is reliable when used for assessing knee pain [26] and in children aged 7 and above [27].

Several secondary outcome measures were also taken. Firstly, the participant’s perception of the impact of the intervention on their condition was measured using the Patient’s Global Impression of Change (PGIC) scale [28]. This 7-point scale, yet to be validated for use with children, ranges from very much worse (7) through no change (4) to very much improved (1). Secondly, the participant’s functional abilities in daily living activities were measured using the Childhood Health Assessment Questionnaire (CHAQ), previously validated in rheumatological conditions [29] and used with hypermobile children [15,30]. Thirdly, the Child Health Questionnaire (CHQ), measuring 12 domains scored from 0 (worst possible health status) to 100 (best possible health status), was used to assess change in parent-reported health-related quality of life. Physical and psychosocial summary scores were then calculated according to user guidelines, where a score of 50 represents the mean of the normative population and one standard deviation is 10 [31]. The CHQ individual domain and summary scores are used extensively within rheumatological literature [32,33] and the Australian adaptation used has been validated [34].

Fourthly, quadriceps and hamstrings muscle strength at neutral and 10° knee hyperextension were measured on both the left and right side using a hand-held dynamometer (Hadland Photonics, Melbourne, Australia). The mean of these 4 measures on each side were used to calculate overall thigh strength. Hand-held dynamometry of the knee extensors and flexors performed with set protocols has been shown to have good reliability and validity in this age range [35]. Finally, the number of flights of stairs the participant could ascend and descend in two minutes was measured, with the participant rating their level of perceived exertion on completion using the OMNI Scale, validated in children within this age range [36]. Stair ascent and descent was chosen to assess function in this population as this challenging task requires greater dynamic knee control and force production in a common activity of daily living [37].

### Group interventions

Following the 2nd assessment, participants were randomly assigned to either exercise programme. The simple randomisation list was generated in a 1:1 ratio using a computer-generated sequence by a person independent of the research group. Treatment allocation was concealed in a sealed, opaque, sequentially numbered envelope which was opened by the treating physiotherapist just prior to the participant’s first physiotherapy session. All physiotherapy sessions were provided by an experienced pediatric physiotherapist who was blinded to the assessment results, and patients were blinded to the difference between the two exercise programmes. Each child received weekly physiotherapy sessions for 4 weeks, followed by fortnightly sessions for 4 weeks, with a total of 6 sessions provided over the 8 week treatment period. Each session lasted 30 – 60 minutes and included review and progression of the exercise programme.

**Figure 1:**
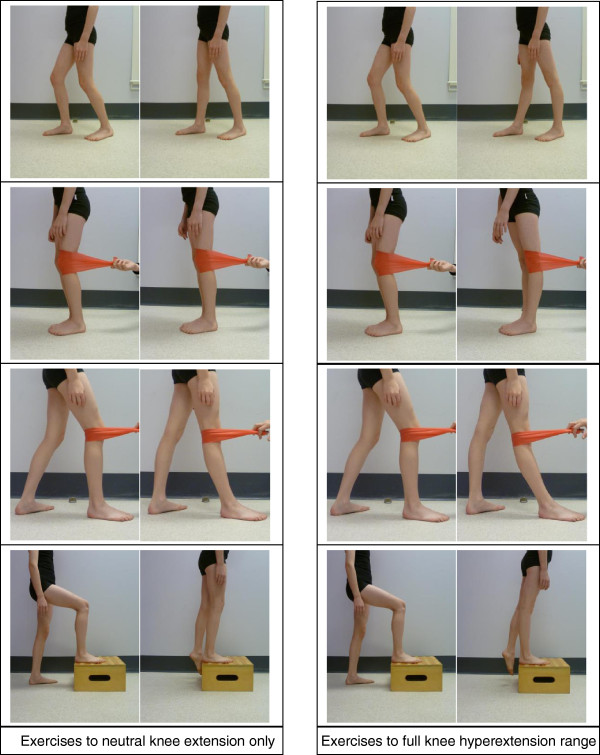
Examples of exercises performed in each treatment group.

The same exercises were provided to each treatment group, with the only difference being the range in which exercises were carried out (Figure 1). Participants in one group were required to perform all exercises to neutral knee extension only, and actively discouraged from moving into any knee hyperextension by verbal prompts and physical blocks. The other group were actively encouraged to exercise into their full knee hyperextension range and positioned for all exercises to allow this. All participants were advised that exercises should be performed painlessly and, if required, modifications were made by the physiotherapist to each exercise to ensure they remained pain-free. The range into which the children exercised was checked by the physiotherapist as the child performed the exercise and reinforced at each session.

The exercise programme consisted of 8 exercises of increasing difficulty, including isometric exercises of the hamstrings and quadriceps muscles in supine, theraband resisted and joint control exercises in standing, eccentric hamstring strengthening in prone, gluteus medius strengthening in side lying and hip abductor strengthening in standing. All participants began with the same basic exercises and as they gained control and competence of each exercise, exercises were progressed by increasing repetitions and resistance and adding more complex exercises on an individual basis. Exercise progression ensured the participant was constantly being challenged while good form was being maintained. Participants were given 3 - 5 exercises within their individual capacity to complete at home a minimum of 5 times per week, and advised they should take no more than 30 minutes per session. This programme was designed to follow the American Academy of Pediatrics’ policy for strength training in children and adolescents [38]. Handouts with pictures depicting the exercises to be performed were given to the participants at each physiotherapy session. Participants and their parents were asked to record their exercise compliance in a diary.

Following the 8 week intervention, participants underwent a third assessment by an assessor blinded to treatment allocation.

### Statistical methods & data analysis

To have 80% power to detect differences as large as one standard deviation (Cohen’s D =1) on each measure at 5% significance, 26 participants (13 per group) were required. A further 3 participants were included to allow for a predicted 10% withdrawal and drop-out rate [39]. Data analysis was performed using SPSS version 19.0 following completion of all assessments. Baseline demographic data was recorded. Body mass index (BMI) centiles were calculated from age and gender specific reference values [40]. Means and standard deviations were calculated for continuous data, and frequencies calculated for categorical data. Baseline demographics were examined for any differences between groups using an unpaired t-test for continuous data and chi-square test for categorical data. Analysis was performed on intention-to-treat principles, using a groups-by-repeated measures ANOVA, with two treatment groups and three measurement occasions. A set of two orthogonal planned contrasts was selected for the three-level factor Time, where contrast one compared the first and second assessment scores in the control period to check for any change in status, and contrast two analysed the effect of training by comparing the final assessment with the mean of the first two assessments. Means and standard deviations are presented for all variables, with 95% confidence intervals calculated, and significance determined at the 0.05 level for secondary measures and at the 0.001 level for primary measures. A unitless effect size measure is represented as Cohen’s D where 0.2 is considered a small effect, 0.5 a moderate and 0.8 a large effect [39].

## Results

**Figure 2:**
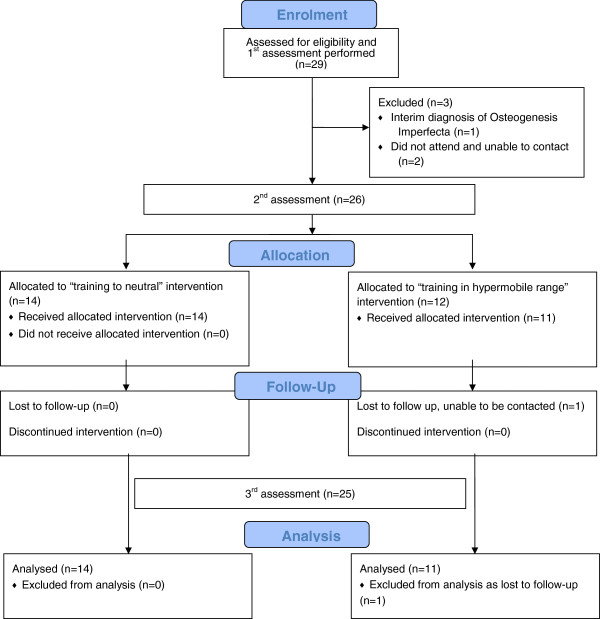
Flow diagram of the trial.

Twenty nine children were recruited for the trial, with 4 drop-outs (Figure 2), including 1 patient excluded following bone densitometry results and further medical assessment resulting in a provisional diagnosis of Osteogenesis Imperfecta. Twenty-five children were then randomly allocated to receiving physiotherapy treatment exercising in either the hypermobile range or only to neutral knee extension. The trial stopped when ethical approval ceased at 5 years. No adverse events, defined as joint injuries including dislocations and subluxations, were reported in either treatment group.

### Baseline data

Table 1 presents the baseline characteristics of each treatment group and combined data. Children in the hypermobile training group were older (p=0.04) with no other statistically significant differences present at baseline. All participants met the Brighton criteria [10] for the diagnosis of JHS. All children reported experiencing knee pain for ≥3 months. Back pain and hand pain when writing, constituted the most common musculoskeletal complaints in addition to knee pain. Most participants were recruited from the Orthopaedic (n=11) and Connective Tissue Dysplasia (n=9) clinics with the remainder from the Sports Medicine (n=5), Physiotherapy (n=3) and Rheumatology (n=1) clinics.

**Table 1:** Baseline characteristics

**Characteristics **^**a**^	**Hypermobile training group (n=12)**	**Neutral training group (n=14)**	**Combined (n=29)†**
Age, years	13.48 (3.05)	11.02 (2.51)*	12.04 (2.93)
Gender: female, *n* (%)	10 (83.3%)	8 (57.1%)	19 (65.5%)
BMI centile	51.25 (26.74)	65.67 (33.11)	61.36 (30.17)
Patient new to service: yes, *n* (%)	11 (91.6%)	10 (71.4%)	24 (82.8%)
Beighton Score (/9)	7.7 (1.0)	6.9 (1.1)	7.14 (1.16)

^a^ Categorical variables: number of participants (%), Continuous variables: mean (S.D).

† Includes drop-outs.

* p<0.05 indicating statistically significant difference between the neutral and training groups.

### Effects of the control period prior to the intervention

Statistical analysis was conducted to check for any changing values during the control period between the first and second assessments. The parent-reported role limitations in emotion and behaviour domain of the CHQ (p=0.02), and consequently the psychosocial summary score (p=0.03), significantly improved during the baseline control period (Additional file 1). No other significant differences were found (all p>0.06).

### Outcome for combined groups

Table 2 presents the effect of the exercise intervention for all participants regardless of group assignment. Statistically significant pre-post improvements were made in thigh strength, child report of knee pain, and parent-reported physical and psychosocial summary scores, whereas there were no significant pre-post changes in the functional measures of the CHAQ or stair ascent/descent. The individual domains of physical functioning, behaviour, bodily pain, self-esteem, mental health and parent emotional impact on the CHQ all showed significant pre-post improvements (Additional file 2).

**Table 2:** Overall effects of exercise training (groups combined n=25)

**Outcome measure**	**Baseline mean (SD)**	**Post-treatment mean (SD)**	**Post-treatment –baseline**			**P value**	**Cohen’s D**
			**Difference between means**	**Lower bound of 95% CI**	**Upper bound of 95% CI**		
**Primary Outcome Measures**							
Child’s report of mean knee pain over the week ^1^	39.4(14.2)	24.2 (18.4)	−14.5	−5.2	−23.8	0.004*	0.89
Child’s report of maximum knee pain over the week ^1^	55.5(18.8)	37.4 (27.5)	−18.1	−6.7	−29.4	0.003*	0.78
**Secondary Outcome Measures**							
*Child Reported Measures*							
PGIC^2^	.23 (1.04)	1.77 (.91)	1.53	1.03	2.04	<0.001*	1.57
CHAQ 38^3^	−0.05 (0.57)	0.02 (0.66)	0.066	−0.11	0.24	0.433	0.11
*Physical Measures*							
Overall thigh strength (N)	4.19 (2.02)	5.25 (1.99)	1.06	0.39	1.72	0.004*	0.53
No. of flights of stairs ran in 2 minutes	18.6 (5.73)	20.33 (5.49)	1.73	−0.48	3.94	0.11	0.29
*Parent Reported Measures (CHQ-PF50)*^*4*^							
Physical Summary Score	37.97 (12.56)	43.31 (11.26)	5.34	1.73	8.94	0.002*	0.45
Psychosocial summary score	48 (10.25)	50.73 (11)	2.73	−0.33	5.8	0.03*	0.26

^1^ Using 2 separate 0 - 100 mm VAS’s, a higher score depicts more pain.

^2^ Patient’s Global Impression of Change (PGIC).

^3^ Child Health Assessment Questionnaire – 38 question version (CHAQ-38).

^4^ Child Health Questionnaire (CHQ).

*p<0.05 statistically significant.

### Comparison of effects of treatment between training groups

**Figure 3:**
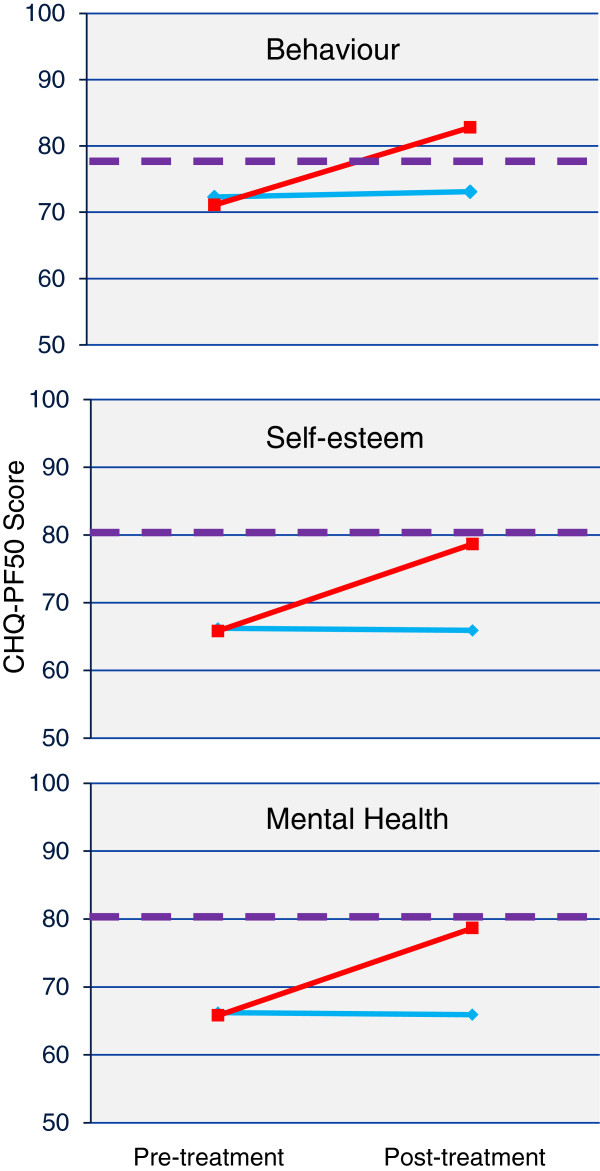
**Improvements in psychosocial measures from exercise training in the hypermobile range.** Legend: red line - Training into the hypermobile range, light blue line - Training into the neutral range, violet broken line - Australian normative value [34].

No significant differences were found between treatment groups in the primary outcome measure of pain, or on any of the secondary child reported or physical measures of strength or function (Table 3). For between-group tests, a significant difference showing better outcome for the group exercising into their hypermobile range was evident in the CHQ psychosocial summary score (p=0.009), whereas the group exercising into their neutral range had statistically significant greater improvements in their physical summary score (p=0.037). Three of the individual domains – self-esteem (p=0.03), behaviour (p=0.019) and mental health (p=0.001) – significantly favoured exercise into the hypermobile range (Figure 3 and Additional file 3). The self-esteem and mental health domains were significantly lower than Australian normative values [34] at baseline (all p<0.05) with only the hypermobile training group equaling Australian norms post treatment (self-esteem: hypermobile p=0.84, neutral p<0.05; mental health: hypermobile p=0.53, neutral p<0.05). There were no statistically significant differences between the mean scores of either training group and the Australian norms pre- or post-training in the behaviour domain (all p>0.05). None of the individual domains significantly favoured the group exercising to the neutral range.

**Table 3:** Comparison of effects of exercise training between treatment groups

**Outcome measure**	**Neutral training group (n=14)**			**Hypermobile training group (n=11)**			**Difference in change scores between groups**			**P value**	**Cohen’s D**
	**Baseline mean (SD)**	**Post-treatment mean (SD)**	**Mean change**	**Baseline mean (SD)**	**Post-treatment mean (SD)**	**Mean change**					
							**Mean difference (H-N)**	**Lower bound of 95% CI**	**Upper bound of 95% CI**		
**Primary Outcome Measures**											
Child’s report of mean knee pain over the week ^1^	40.04 (16.59)	20.14 (18.37)	−19.9	38.55 (16.89)	29.36 (17.99)	−9.19	10.71	−7.9	29.33	0.246	0.61
Child’s report of maximum knee pain over the week ^1^	57.68 (23.12)	35.64 (28.57)	−22.04	53.23 (23.55)	39.18 (27.21)	−14.05	7.99	−14.66	30.64	0.473	0.31
**Secondary Outcome Measures**											
*Child Reported Measures*											
PGIC^2^	0.29 (1.14)	1.71 (0.99)	1.43	0.18 (0.87)	1.82 (0.75)	1.64	0.21	−0.81	1.22	0.675	0.22
CHAQ 38^3^	−0.13 (0.44)	−0.01 (0.60)	0.12	0.04 (0.71)	0.05 (0.72)	0.02	0.10	−0.25	0.45	0.552	0.16
*Physical Measures*											
Overall thigh strength (N)	4.02 (1.72)	4.9 (2.17)	0.88	4.38 (2.37)	5.59 (1.45)	1.21	0.33	−1.66	1	0.608	0.17
No. of flights of stairs ran in 2 minutes	16.32 (5.00)	20.11 (5.52)	3.79	20.88 (6.69)	20.55 (5.44)	−0.33	−4.12	0.301	−8.523	0.118	0.73
*Parent Reported Measures (CHQ-PF50)*^4^											
Physical Summary Score	32.01 (11.86)	42.08 (10.81)	10.07	41.61 (14.96)	43.91 (15.05)	2.3	−7.77	−14.99	−0.55	0.037*	0.59
Psychosocial Summary Score	46.35 (12.26)	45.41 (13.49)	−0.94	46.29 (8.95)	54.41 (4.42)	8.12	9.06	2.66	15.47	0.009*	0.83

^1^ Using the 0 - 100 mm VAS.

^2^ Patient’s Global Impression of Change (PGIC).

^3^ Child Health Assessment Questionnaire – 38 question version (CHAQ-38).

^4^ Child Health Questionnaire (CHQ).

*p<0.05 statistically significant.

## Discussion

Joint pain is the most common complaint in children with JHS [8] and pain severity was therefore chosen as the primary outcome measure in this trial. Large effect sizes and statistically significant differences were found following the exercise period. Child reported mean knee pain decreased by 36%, maximum knee pain decreased by 32%, and parent-reported bodily pain improved by 37%. A 30% improvement in pain scores is considered clinically significant in other rheumatological conditions [41] and these improvements are similar to those found previously when assessing the efficacy of exercise for children with JHS [15]. As the treatment was provided to children and adolescents of both genders across ages 7-16 years, the results suggest that this programme may provide similar pain reductions in pain intensity for other children and adolescents with JHS and knee pain seen within the Australian healthcare setting. To provide a more comprehensive understanding of the impact of physiotherapy based interventions on the child’s overall pain experience, future research should also assess pain frequency and duration.

As knee hyperextension may result in infrapatellar fat pad, or anterior capsule impingement eliciting pain, it is possible that the children exercising to full hyperextension may have had greater difficulties performing the exercise programme pain-free. Consequently, those patients who actively limited exercises to neutral knee extension only may have gained greater physical benefits from the exercise programme over the 8-week period. It may be appropriate when managing these patients to exercise first to neutral knee extension until symptoms settle, before challenging their motion control in the hyperextension range. Given the physical benefits of an exercise to neutral paradigm and the psychosocial benefits of an exercise into hypermobile exercise paradigm, we propose that clinicians begin with the neutral, and progress to the hypermobile range, to achieve a more holistic outcome. This graded approach to exercise in JHS has been previously proposed by a group of expert therapists [42]. Rate of exercise progression and use of analgesia was not measured within this study, but would be worthwhile to consider in future research.

Considering the overall improvement when results of the two training groups were combined, moderate effect sizes were demonstrated in all other statistically significant findings. The effect sizes observed here suggests that studies with larger sample sizes would be worthwhile.

Parent ratings of a child’s behaviour, self-esteem and mental health are important indicators of their perception of their child’s psychosocial wellbeing. These measures were specifically influenced by an exercise program that took the child’s limbs into hypermobile range, over and above the general effects of exercise shared by both groups. The improvements seen in these domains were not only significantly different between groups, but also demonstrated that exercising into the hypermobile range significantly improved self-esteem and mental health levels to equal that of Australian normative values [34]. The reason for this finding may be due to a shift in parental perception of their child’s condition contingent on them exercising into hypermobile range. Children and parents were only aware that two different exercise programmes were being studied and were blinded to the range differences. Having the physiotherapist encourage movement within the hypermobile range may have “normalised” the parent’s perception of their child’s everyday movements that they previously considered unusual and undesirable. The consequent impact on perception of their hypermobility may explain improvements in these psychosocial measures. In light of the known incidence of anxiety disorders with the adult JHS population [43], further research on the mechanism behind this finding is warranted, with the inference that measurement of the child’s own perception of their self-esteem and mental health would be worthwhile.

In contrast to the improvements in psychosocial measures, overall parent-rated physical function improvements favoured those children exercising to only neutral knee extension. Despite strict randomisation, the neutral training group were significantly younger, contrasting to the known effect of strength trainability increasing linearly with age [44]. However, this result may have been due to a ceiling effect, as despite strict randomisation, the group exercising to neutral knee extension had significantly lower physical summary scores at baseline, while final summary scores for each group were similar. Future research with larger study samples would provide definitive results as to whether limiting exercises only to the neutral range does result in measurable differences in physical function outcome.

The CHAQ showed minimal effect from the training intervention and this may be due to the global nature of the questionnaire. Questions included those related to fine motor function and daily activities such as eating and dressing which would not be expected to change when undertaking an exercise programme aimed only at knee joint function. This provides important methodological considerations for future studies in this patient population as the use of a functional measure for children more specific to the knee joint might have been more sensitive to change. Recently, the Knee Injury and Osteoarthritis Outcome score has been modified for children (KOOS-Child) [45] and the Modified International Knee Documentation Committee Subjective Knee Form has demonstrated acceptable psychometric properties for use in children with a variety of knee disorders (Pedi-IKDC) [46]. The use of one of these new measures is likely to be more appropriate in this population.

Similarly, no significant changes were seen in the child’s ability to climb flights of stairs in 2 minutes, most likely as a result of a ceiling effect. Each flight consisted of 12 stairs and even when running, no greater than 25 flights was ever achieved in the 2 minutes allowed. This provided minimal opportunity for improvement in many of the participants.

Significant moderate sized improvements in muscle strength resulted from training regardless of group allocation. As expected, as both training groups were exercising to the same intensity and duration, no differences were evident between groups.

Knee joint proprioception has previously been shown to be reduced in children with JHS [18] and exercise programmes in adults with JHS have demonstrated improvements in proprioception as a result of exercise training [14]. The inclusion of knee joint proprioception as an outcome measure for this study may have provided further insight into the mechanisms by which pain intensity and function gained improvements.

No significant differences were found during the baseline period with the exception of parent-reported role limitations in emotion and behaviour and hence the psychosocial summary score. We hypothesise that the improvement in the child’s behaviour may have occurred as a result of enrolment into the trial. The first assessment occurred at this point and children met with a specialist in the area who acknowledged their symptoms and hypermobility as the cause of it, and provided reassurance that physiotherapy would help to improve their pain. Within this cohort, 83% of the children were previously unknown to the multidisciplinary hypermobility service at our centre. Despite all children having GJH and significant knee pain (mean 39.4/100 on the VAS over the previous week), these children had not previously been recognised as having JHS. Delayed diagnosis of this condition has previously been reported in the literature [8] and this delay may contribute to provision of less than optimal management [47].

A multi-system disorder, JHS has significant adverse impacts on the affected child and their family’s daily functioning. Adib et al [8] reported 41% of children with JHS miss important periods of schooling and 67% experience limitations in their physical activities as a result of their symptoms. Children with JHS and knee pain also experience significantly reduced quality of life compared to their healthy peers [48]. There is therefore a critical need for empirically-based evidence to guide the management of this condition.

The present study is the first RCT comparing the effectiveness of performing individualised and progressive exercises either to neutral or into the full hypermobile range of motion for individuals with symptomatic hypermobility, and the second RCT on the effectiveness of physiotherapy management for children with JHS. It provides further evidence to support the use of physiotherapy management, however because no long term follow-up was undertaken within this study, it remains unknown if the effect of the intervention washes out. The impact of this intervention on medication use and participation in daily activities such as school attendance and physical activities also warrants further investigation.

## Conclusions

This study has demonstrated that a physiotherapist-supervised exercise programme is significantly effective in reducing pain, improving health-related quality of life, and increasing muscle strength in children with JHS and knee pain. In addition, these exercises were found to be more effective in improving the child’s self-esteem, mental health and behaviour when performed into the full hypermobile range rather than when performed only to neutral knee extension. Conversely, parent-reported overall physical health significantly favoured exercising only to neutral extension.

## Abbreviations

JHS: Joint hypermobility syndrome; GJH: Generalised joint hypermobility; RCT: Randomised controlled trial; VAS: Visual analogue scale; PGIC: Patients’ global impression of change; CHAQ: Child health assessment questionnaire; CHQ: Child health questionnaire.

## Competing interests

The authors declare that they have no competing interests.

## Authors’ contributions

VP was involved in the study conception and design, data acquisition and analysis. LT was involved in study conception and design, data acquisition and analysis. RA was involved in study design, data analysis and interpretation. CM was involved in data acquisition and interpretation. LN was involved in study design, data analysis and interpretation. All authors were involved in manuscript preparation and have read and approved the final manuscript.

## Supplementary Material

Additional file 1 Effects of the control period prior to the intervention (n=26): All outcome measures. Click here for file

Additional file 2 Overall effects of exercise training (groups combined n=25): Individual domains of the CHQ-PF50. Click here for file

Additional file 3 Comparison of effects of training between treatment groups (neutral n=14, hypermobile n=11): Individual domains of the CHQ-PF50. Click here for file

## References

[B1] Clinch J Deere K Sayers A Palmer S Riddoch C Tobias JH Clark EM Epidemiology of generalized joint laxity (hypermobility) in fourteen-year-old children from the UK: a population-based evaluation Arthritis & Rheumatism 2011 63 2819 2827 10.1002/art.30435 21547894 PMC3164233

[B2] Seckin U Tur BS Yilmaz O Yagci I Bodur H Arasil T The prevalence of joint hypermobility among high school students Rheumatol Int 2005 25 260 263 10.1007/s00296-003-0434-9 14745505

[B3] El-Garf AK Mahmoud GA Mahgoub EH Hypermobility among Egyptian children: prevalence and features J Rheumatol 1998 25 1003 1005 9598908

[B4] Cheng JC Chan PS Hui PW Joint laxity in children J Pediatr Orthop 1991 11 752 756 10.1097/01241398-199111000-00010 1960200

[B5] Leone V Tornese G Zerial M Locatelli C Ciambra R Bensa M Pocecco M Joint hypermobility and its relationship to musculoskeletal pain in schoolchildren: a cross-sectional study Arch Dis Child 2009 94 627 632 10.1136/adc.2008.150839 19465584

[B6] Hasija RP Khubchandani RP Shenoi S Joint hypermobility in Indian children Clin Exp Rheumatol 2008 26 146 150 18328164

[B7] Juul-Kristensen B Kristensen JH Frausing B Jensen DV Rogind H Remvig L Motor competence and physical activity in 8-year-old school children with generalized joint hypermobility Pediatrics 2009 124 1380 1387 10.1542/peds.2009-0294 19822597

[B8] Adib N Davies K Grahame R Woo P Murray KJ Joint hypermobility syndrome in childhood. A not so benign multisystem disorder? Rheumatol (Oxford) 2005 44 744 750 10.1093/rheumatology/keh557 15728418

[B9] El-Metwally A Salminen JJ Auvinen A Macfarlane G Mikkelsson M Risk factors for development of non-specific musculoskeletal pain in preteens and early adolescents: a prospective 1-year follow-up study BMC Musculoskelet Disord 2007 8 46 10.1186/1471-2474-8-46 17521435 PMC1891107

[B10] Grahame R Bird HA Child A The revised (Brighton 1998) criteria for the diagnosis of benign joint hypermobility syndrome (BJHS) J Rheumatol 2000 27 1777 1779 10914867

[B11] Schubert-Hjalmarsson E Ohman A Kyllerman M Beckung E Pain, balance, activity, and participation in children with hypermobility syndrome Pediatr 2012 24 339 344 10.1097/PEP.0b013e318268e0ef 22965207

[B12] Tinkle BT Bird HA Grahame R Lavallee M Levy HP Sillence D The lack of clinical distinction between the hypermobility type of Ehlers-Danlos syndrome and the joint hypermobility syndrome (a.k.a. hypermobility syndrome) Am J Med Genet A 2009 149A 2368 2370 10.1002/ajmg.a.33070 19842204

[B13] Murray KJ Hypermobility disorders in children and adolescents Best Pract Res Clin Rheumatol 2006 20 329 351 10.1016/j.berh.2005.12.003 16546060

[B14] Ferrell WR Tennant N Sturrock RD Ashton L Creed G Brydson G Rafferty D Amelioration of symptoms by enhancement of proprioception in patients with joint hypermobility syndrome Arthritis Rheum 2004 50 3323 3328 10.1002/art.20582 15476239

[B15] Kemp S Roberts I Gamble C Wilkinson S Davidson JE Baildam EM Cleary AG McCann LJ Beresford MW A randomized comparative trial of generalized vs targeted physiotherapy in the management of childhood hypermobility Rheumatol (Oxford) 2010 49 315 325 10.1093/rheumatology/kep362 19948753

[B16] Castori M Morlino S Celletti C Celli M Morrone A Colombi M Camerota F Grammatico P Management of pain and fatigue in the joint hypermobility syndrome (a.k.a. Ehlers-Danlos syndrome, hypermobility type): principles and proposal for a multidisciplinary approach Am J Med Genet A 2012 158A 2055 2070 10.1002/ajmg.a.35483 22786715

[B17] Gedalia A Brewer EJ Joint hypermobility in pediatric practice–a review J Rheumatol 1993 20 371 374 8474078

[B18] Fatoye F Palmer S Macmillan F Rowe P van der Linden M Proprioception and muscle torque deficits in children with hypermobility syndrome Rheumatol (Oxford) 2009 48 152 157 10.1093/rheumatology/ken435 19088133

[B19] Fatoye FA Palmer S van der Linden ML Rowe PJ Macmillan F Gait kinematics and passive knee joint range of motion in children with hypermobility syndrome Gait Posture 2011 33 447 451 10.1016/j.gaitpost.2010.12.022 21300548

[B20] Keer R SRP MSMM, Rodney Grahame CMFF, Rosemary K Chapter 6 - Physiotherapy assessment of the hypermobile adult Hypermobility Syndrome 2003 Oxford: Butterworth-Heinemann 67 86

[B21] Keer R Simmonds J Joint protection and physical rehabilitation of the adult with hypermobility syndrome Curr Opin Rheumatol 2011 23 131 136 10.1097/BOR.0b013e328342d3af 21252682

[B22] Russek LN Examination and treatment of a patient with hypermobility syndrome Phys Ther 2000 80 386 398 10758523 10.1093/ptj/80.4.386

[B23] Beighton P Solomon L Soskolne CL Articular mobility in an African population Ann Rheum Dis 1973 32 413 418 10.1136/ard.32.5.413 4751776 PMC1006136

[B24] Castori M Sperduti I Celletti C Camerota F Grammatico P Symptom and joint mobility progression in the joint hypermobility syndrome (Ehlers-Danlos syndrome, hypermobility type) Clin Exp Rheumatol 2011 29 998 1005 22208933

[B25] Tofts LJ Elliott EJ Munns C Pacey V Sillence DO The differential diagnosis of children with joint hypermobility: a review of the literature Pediatr Rheumatol Online J 2009 7 1 10.1186/1546-0096-7-1 19123951 PMC2628911

[B26] Crossley KM Bennell KL Cowan SM Green S Analysis of outcome measures for persons with patellofemoral pain: which are reliable and valid? Arch Phys Med Rehabil 2004 85 815 822 10.1016/S0003-9993(03)00613-0 15129407

[B27] Shields BJ Palermo TM Powers JD Grewe SD Smith GA Predictors of a child’s ability to use a visual analogue scale Child Care Health Dev 2003 29 281 290 10.1046/j.1365-2214.2003.00343.x 12823333

[B28] Farrar JT Young JP LaMoreaux L Werth JL Poole RM Clinical importance of changes in chronic pain intensity measured on an 11-point numerical pain rating scale Pain 2001 94 149 158 10.1016/S0304-3959(01)00349-9 11690728

[B29] Nugent J Ruperto N Grainger J Machado C Sawhney S Baildam E Davidson J Foster H Hall A Hollingworth P The British version of the childhood health assessment questionnaire (CHAQ) and the child health questionnaire (CHQ) Clin Exp Rheumatol 2001 19 S163 S167 11510323

[B30] Ruperto N Malattia C Bartoli M Trail L Pistorio A Martini A Ravelli A Functional ability and physical and psychosocial well-being of hypermobile schoolchildren Clin Exp Rheumatol 2004 22 495 498 15301252

[B31] Landgraf JM Abetz L Ware JE The CHQ User’s Manual 1996 Boston: The Health Institute, New England Medical Centre

[B32] Oliveira S Ravelli A Pistorio A Castell E Malattia C Prieur AM Saad-Magalhães C Murray KJ Bae SC Joos R Proxy-reported health-related quality of life of patients with juvenile idiopathic arthritis: the pediatric rheumatology international trials organization multinational quality of life cohort study Arthritis Rheum 2007 57 35 43 10.1002/art.22473 17266064

[B33] Ruperto N Pistorio A Ravelli A Hasija R Guseinova D Filocamo G Demirkaya E Malattia C Martini A Criteria to define response to therapy in paediatric rheumatic diseases Eur J Clin Pharmacol 2011 67 Suppl 1 125 131 21103985 10.1007/s00228-010-0937-8

[B34] Waters E Salmon L Wake M Hesketh K Wright M The child health questionnaire in Australia: reliability, validity and population means Aust N Z J Public Health 2000 24 207 210 10.1111/j.1467-842X.2000.tb00145.x 10790944

[B35] Hébert LJ Maltais DB Lepage C Saulnier J Crête M Perron M Isometric muscle strength in youth assessed by hand-held dynamometry: a feasibility, reliability, and validity study Pediatr Phys Ther 2011 23 289 299 10.1097/PEP.0b013e318227ccff 21829128

[B36] Utter AC Robertson RJ Nieman DC Kang J Children’s OMNI scale of perceived exertion: walking/running evaluation Med Sci Sports Exerc 2002 34 139 144 10.1097/00005768-200201000-00021 11782659

[B37] Bovi G Rabuffetti M Mazzoleni P Ferrarin M A multiple-task gait analysis approach: Kinematic, kinetic and EMG reference data for healthy young and adult subjects Gait Posture 2011 33 6 13 10.1016/j.gaitpost.2010.08.009 21123071

[B38] McCambridge TM Stricker PR Strength training by children and adolescents Pediatrics 2008 121 835 840 18381549 10.1542/peds.2007-3790

[B39] Cohen J A power primer Psychol Bull 1992 112 155 159 19565683 10.1037//0033-2909.112.1.155

[B40] Kuczmarski RJ Ogden CL Grummer-Strawn LM Flegal KM Guo SS Wei R Mei Z Curtin LR Roche AF Johnson CL CDC growth charts: United States Advance data 2000 314 1 27 11183293

[B41] Giannini EH Ruperto N Ravelli A Lovell DJ Felson DT Martini A Preliminary definition of improvement in juvenile arthritis Arthritis Rheum 1997 40 1202 1209 9214419 10.1002/1529-0131(199707)40:7<1202::AID-ART3>3.0.CO;2-R

[B42] Simmonds JV Keer RJ Hypermobility and the hypermobility syndrome, part 2: assessment and management of hypermobility syndrome: illustrated via case studies Manual Ther 2008 13 e1 e11 10.1016/j.math.2007.11.001 18221908

[B43] Bulbena A Gago J Pailhez G Sperry L Fullana MA Vilarroya O Joint hypermobility syndrome is a risk factor trait for anxiety disorders: a 15-year follow-up cohort study Gen Hosp Psychiatry 2011 33 363 370 10.1016/j.genhosppsych.2011.03.004 21762833

[B44] Behringer M Vom Heede A Yue Z Mester J Effects of resistance training in children and adolescents: a meta-analysis Pediatrics 2010 126 e1199 e1210 10.1542/peds.2010-0445 20974785

[B45] Ortqvist M Roos EM Brostrom EW Janarv PM Iversen MD Development of the Knee Injury and Osteoarthritis Outcome Score for children (KOOS-Child): comprehensibility and content validity Acta orthopaedica 2012 83 666 673 10.3109/17453674.2012.747921 23140110 PMC3555443

[B46] Kocher MS Smith JT Iversen MD Brustowicz K Ogunwole O Andersen J Yoo WJ McFeely ED Anderson AF Zurakowski D Reliability, validity, and responsiveness of a modified international knee documentation committee subjective knee form (Pedi-IKDC) in children with knee disorders Am J Sports Med 2011 39 933 939 10.1177/0363546510383002 21068443

[B47] Ross J Grahame R Joint hypermobility syndrome Br Med J 2011 342 c7167 10.1136/bmj.c7167 21252103

[B48] Fatoye F Palmer S Macmillan F Rowe P van der Linden M Pain intensity and quality of life perception in children with hypermobility syndrome Rheumatol Int 2012 32 1277 1284 10.1007/s00296-010-1729-2 21267571

