# Supplementary material for: Exercise in children with joint hypermobility syndrome and knee pain: a randomised controlled trial comparing exercise into hypermobile versus neutral knee extension
Source: Pediatr Rheumatol Online J. 2013 Aug 14;11:30. doi: 10.1186/1546-0096-11-30 (PMC3751568; doi:10.1186/1546-0096-11-30)
Supplement: Additional file 1 — Effects of the control period prior to the intervention (n=26): All outcome measures. [file 1546-0096-11-30-S1.pdf]

Outcome measure	First assessment mean (SD) (n=26)	Second assessment mean (SD) (n=26)	p-value
**Primary Outcome Measures**			
Child’s report of mean knee pain over the week [1]	40.58 (18.97)	38.01 (14.74)	0.47
Child’s report of maximum knee pain over the week [1]	58.06 (23.72)	52.85 (23.26)	0.34
**Secondary Outcome Measures**			
Child Reported Measures			
PGIC Change[2]	N/A	0.23 (1.04)	N/A
CHAQ 38[3]	-0.04 (0.57)	-0.05 (0.59)	0.86
Overall lower limb strength (N)	3.90 (2.03)	4.48 (2.29)	0.08
No. of flights of stairs ran in 2 minutes	17.93 (5.53)	19.26 (6.19)	0.06
Parent Reported Measures (CHQ-PF50)[4]			
Physical Summary Score	36.28 (15.65)	37.34 (11.20)	0.65
Psychosocial Summary Score	44.90 (11.60)	47.73 (10.89)	0.03*
Physical Functioning	70.49 (24.2)	72.94 (20.08)	0.41
Role limitations – emotional and behavioural	76.39 (30.17)	89.58 (20.51)	0.02*
Role limitations - physical	71.53 (33.34)	69.79 (31.60)	0.78
Bodily Pain	39.79 (23.17)	44.58 (16.12)	0.28
Behaviour	70.34 (17.25)	73.06 (13.06)	0.23
Mental Health	70.42 (17.58)	71.77 (16.31)	0.48
Self Esteem	66.87 (19.21)	65.13 (21.93)	0.55
General Health	63.82 (20.20)	64.66 (16.92)	0.77
Parental Emotional Impact	53.93 (28.54)	60.12 (28.94)	0.34
Parental Time Impact	80.16 (26.69)	86.51 (20.55)	0.09
Family Activities	68.11 (28.95)	72.19 (26.96)	0.20

Family Cohesion	67.02 (28.09)	75.12 (23.07)	0.14

[1] Using 2 separate 0 - 100mm VAS's, a higher score depicts more pain

[2] Patient's Global Impression of Change (PGIC)

[3] Child Health Assessment Questionnaire – 38 question version (CHAQ-38)

[4] Child Health Questionnaire (CHQ)

*p<0.05 statistically significant
