# Supplementary material for: Exercise in children with joint hypermobility syndrome and knee pain: a randomised controlled trial comparing exercise into hypermobile versus neutral knee extension
Source: Pediatr Rheumatol Online J. 2013 Aug 14;11:30. doi: 10.1186/1546-0096-11-30 (PMC3751568; doi:10.1186/1546-0096-11-30)
Supplement: Additional file 2 — Overall effects of exercise training (groups combined n=25): Individual domains of the CHQ-PF50. [file 1546-0096-11-30-S2.pdf]

Outcome measure	Baseline mean (SD)	Post-treatment mean (SD)	Post-treatment –Baseline			P value	Cohen's D
			Difference between means	Lower bound of 95% CI	Upper bound of 95% CI		
Physical Functioning	71.71 (21.7)	81.04 (19.8)	9.33	2.74	15.92	0.008*	0.45
Role limitations – emotional and behavioural	82.99 (24.83)	88.43 (20.82)	5.44	-6.8	17.68	0.363	0.24
Role limitations - physical	70.66 (31.82)	77.08 (29.0)	6.42	-5.26	18.1	0.263	0.21
Bodily Pain	42.19 (19.25)	57.92 (18.99)	15.73	25.23	6.23	0.003*	0.82
Behaviour	71.7 (14.85)	77.95 (18.49)	6.25	1.8	10.7	0.009*	0.37
Mental Health	71.09 (16.6)	76.25 (14.91)	5.16	1.31	9.99	0.011*	0.33
Self Esteem	66.00 (20.04)	72.28 (15.48)	6.28	-0.26	-12.3	0.042*	0.34
General Health	64.24 (18.33)	67.66 (17.18)	3.42	-4.22	11.05	0.359	0.19
Parental Emotional Impact	57.02 (28.29)	69.46 (24.02)	12.44	2.51	22.37	0.017*	0.46
Parental Time Impact	83.33 (23.24)	85.48 (21.15)	2.15	-2.58	6.87	0.349	0.10
Family Activities	70.15 (27.51)	72.32 (28.41)	2.17	-6.47	10.81	0.600	0.08
Family Cohesion	71.07 (25.38)	70.64 (17.1)	-0.54	-8.04	7.17	0.904	0.03

*p<0.05 statistically significant
