# Supplementary figures and images for: Exercise in children with joint hypermobility syndrome and knee pain: a randomised controlled trial comparing exercise into hypermobile versus neutral knee extension
Source: Pediatr Rheumatol Online J. 2013 Aug 14;11:30. doi: 10.1186/1546-0096-11-30 (PMC3751568; doi:10.1186/1546-0096-11-30)

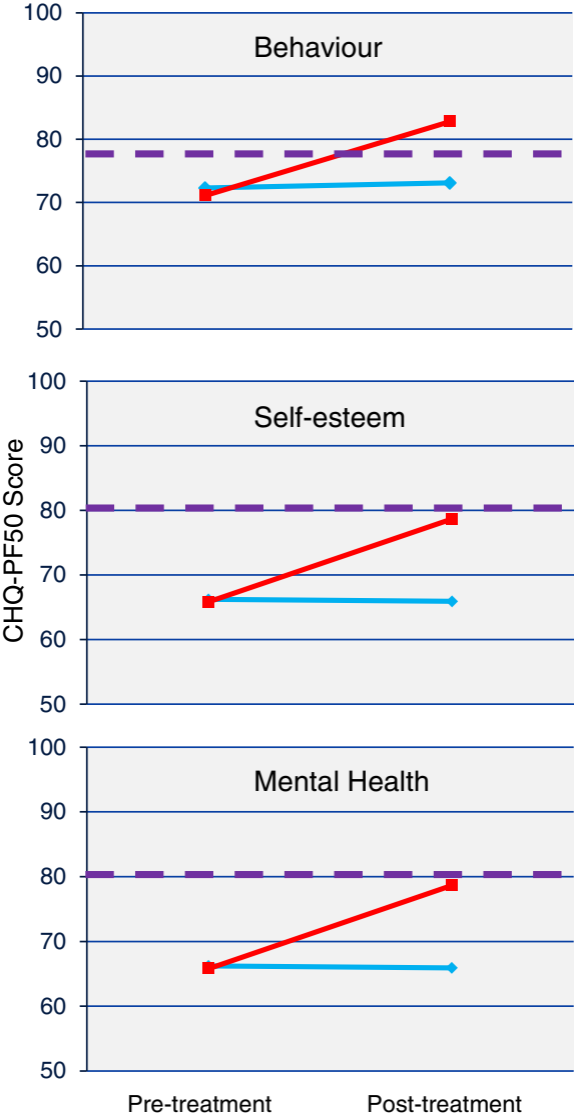

Supplement: Additional file 3 — Comparison of effects of training between treatment groups (neutral n=14, hypermobile n=11): Individual domains of the CHQ-PF50. [file 1546-0096-11-30-S3.pdf]
